# Landscape correlates of space use in the critically endangered African wild dog *Lycaon pictus*

**DOI:** 10.1371/journal.pone.0212621

**Published:** 2019-03-22

**Authors:** Mariëtte E. Pretorius, Nimmi Seoraj-Pillai, Neville Pillay

**Affiliations:** 1 School of Animals, Plants and Environmental Sciences, University of the Witwatersrand, Johannesburg, South Africa; 2 Department of Nature Conservation, Tshwane University of Technology, Pretoria, South Africa; University of Tasmania, AUSTRALIA

## Abstract

Human-carnivore conflict can threaten human life and livelihoods, leading to retaliation that negatively affects carnivore conservation. The endangered African wild dog *Lycaon pictus* is prone to human-carnivore conflict. Therefore, it is imperative to understand which landscape features are associated with African wild dog occurrence since selection or avoidance of these features could predict the levels of conflict. We investigated resource selection in the African wild dog in relation to four anthropogenic landscape features (livestock density, agriculture, roads and human land use) within the landscape that may pose a mortality risk, as well as one natural feature (nature reserves). We compared spatio-temporal space use patterns of four African wild dog packs in north-eastern South Africa. Data were collected from one collared individual per pack. These packs constituted approximately 10% of the total remaining African wild dog population in South Africa. Two packs occurred outside of the Kruger National Park and had access to multiple areas with farmland and other anthropogenic features, whereas the remaining two packs mainly occurred within the boundaries of the Kruger National Park but made occasional forays outside of park boundaries. Utilising Resource Selection Functions and GIS analyses, we found that agricultural landscape features, roads and nature reserves were important predictors of African wild dog occurrence for all four packs. In addition to potential conflict with farmers, high odds of occurrence on roads with fast-moving traffic and road mortality was highlighted as a concern for three of the packs. While farms and areas that house livestock were readily available, pack presence occurred in areas with few farms and low livestock densities, pointing to avoidance of areas where human-carnivore conflict and resulting mortality could occur. Our study highlights potential threats to the persistence of the African wild dog, which can be used to inform future conservation efforts of the species.

## Introduction

Global expansions of anthropogenic activity have increased the occurrence and intensity of human-carnivore conflict and carnivore persecution worldwide [1]. Human-carnivore conflict occurs when the resource demands of humans and carnivores overlap, creating competition for food, space and water [1, 2]. Human-carnivore conflict occurs when carnivores damage poultry, livestock and farmed game and jeopardise human safety [2]. Afflicted people can then retaliate or deliberately persecute conservation priority species [2, 3]. Anthropogenic activities and retaliatory killings have been suggested as the primary drivers of several carnivore declines [4].

Reducing the impact on carnivores in conflicts requires a multi-pronged approach to address the concerns of people and the vulnerability of carnivores. An important first step is to identify the factors that exacerbate conflict, such as the use or avoidance of resources by carnivores. The degree of mortality risk within an environment influences an animal’s decision to remain and use a resource (e.g. food, shelter), or to leave and search elsewhere [5]. The resource selection of an animal describes the quantity of a particular resource used versus the availability of that resource in the habitat [6]. By altering its space use and resource selection, an animal may avoid potentially deadly encounters with conspecifics, other species and humans, and seek out areas of low risk, called refuges [7, 8]. For example, in Botswana, African lion *Panthera leo* mostly avoided cattle *Bos taurus* posts, but when they did use these areas, they travelled at high speeds in order to reduce time spent in these areas and avoided these areas at times of day when humans were most active [8].

We investigated resource use in the African wild dog *Lycaon pictus* to assess whether anthropogenic landscape features that may pose a mortality risk for this endangered canid [8] affected their space use. Despite extensive conservation efforts and protection under the South African National Environmental Management: Biodiversity Act [9], African wild dog populations have been declining dramatically in the past century. Reports indicate low population densities of 2–27 individuals per 1000 km^2^ across a variety of different sub-Saharan ecosystems [10]. Recent population numbers in protected areas in South Africa are estimated to be between 466–554 individuals [11, 12], with one of the few viable populations of approximately 17 packs totalling 250 individuals occurring in the Kruger National Park (hereafter KNP) [10, 13]. Free-roaming African wild dogs outside of reserves in South Africa are estimated at 24 individuals [11].

The decline of the African wild dog has been attributed to multiple factors, including interspecific competition [14], kleptoparasitism [15] and infectious diseases [16]. Mortality on roads carrying heavy traffic poses another danger to these species. For example, nine wild dog individuals were killed on roads in the Greater Mapungubwe Transfrontier Conservation Area, southern Africa within a three-month period in 2012 [17]. Shrinking habitats, conflict with humans and persecution are also ascribed as the major reasons for the drastic population decline [18, 19]. In our study, we focussed on whether road mortality and persecution by farmers are possible reasons for their decline.

African wild dogs are often persecuted by farmers for their alleged depredation of livestock [20, 21] and captive-bred trophy game species [22]. Human-carnivore conflict over captive-bred trophy game species is especially prevalent in South Africa, where a thriving hunting industry on 12000 game farms generates approximately US$ 500 million through annual biltong (dried, cured meat) and trophy hunt sales [23]. Doubt exists as to whether wild dogs are the avid depredators as suggested because they have been shown to avoid human settlements, activities and livestock [24]. Hence, it is important to investigate whether actual resource selection patterns of African wild dog packs reflect their supposed livestock depredating behaviour, as well as which landscape features determine their occurrence. Research on resource selection patterns of African wild dogs is rare. We are aware of only one study that considered the relationship between resource selection patterns and individual behavioural state (e.g. foraging, resting and travelling)[25].

We investigated the resource selection of four wild dog packs in South Africa. Two packs were free ranging outside of formally protected reserves and the remaining two packs were located in the KNP. The two KNP packs were included in our analyses because, although this reserve is fenced, these fences are regularly destroyed by bull elephants *Loxodonta africana* and are therefore porous to animal movements into the neighbouring human-inhabited areas [26]. We assessed the occurrence of the packs in relation to different anthropogenic features that may pose a mortality risk, such as retaliatory killings by livestock farmers or vehicular collisions on major roads. We predicted that the two packs outside of the protected areas would encounter a higher number of anthropogenic landscape features but would avoid these areas as they pose possible mortality risks. Moreover, packs outside of reserves would avoid roads because of the risk of collision with vehicles. Alternatively, roads (as a linear landscape feature) may serve as an attractant to wild dogs as travel routes.

## Materials and methods

The African wild dog data were obtained through a data-sharing collaboration (Ref. DSA1-000) with the Endangered Wildlife Trust (EWT): Carnivore Conservation Programme (CCP). The CCP is a registered project with South African National Parks (SANParks) (Ref. DMOHT804) and the ethics approval to conduct wild dog darting and collaring formed part of the project registration, facilitated by the SANParks Veterinary Wildlife Services under the following Standard Operating Procedures: a) 17/Pr-CSD/SOP/capture, transport, holding facilities (04–17) vs 1; and b) 17/Pr-CSD/pro/tracking + marking (12/16) v1. The welfare and safety of the animals during immobilisation events were also the responsibility of the SANParks Veterinary Wildlife Services (standard operating procedures for the capture, transportation and maintenance in holding facilities of wildlife). The EWT Limpopo field team operated under the following Threatened or Protected Species (TOPS) Regulations permits under NEMBA permit numbers: OWM 974/2012, OWM 1297/2013. The darting in Limpopo was conducted by Dr Peter Caldwell who is a TOPS registered veterinary surgeon. A waiver from the Animal Research Ethics Committee of the University of the Witwatersrand was obtained to cover the use of data obtained (Ref. no: AREC-101210-002).

### Study area

**Fig 1:**
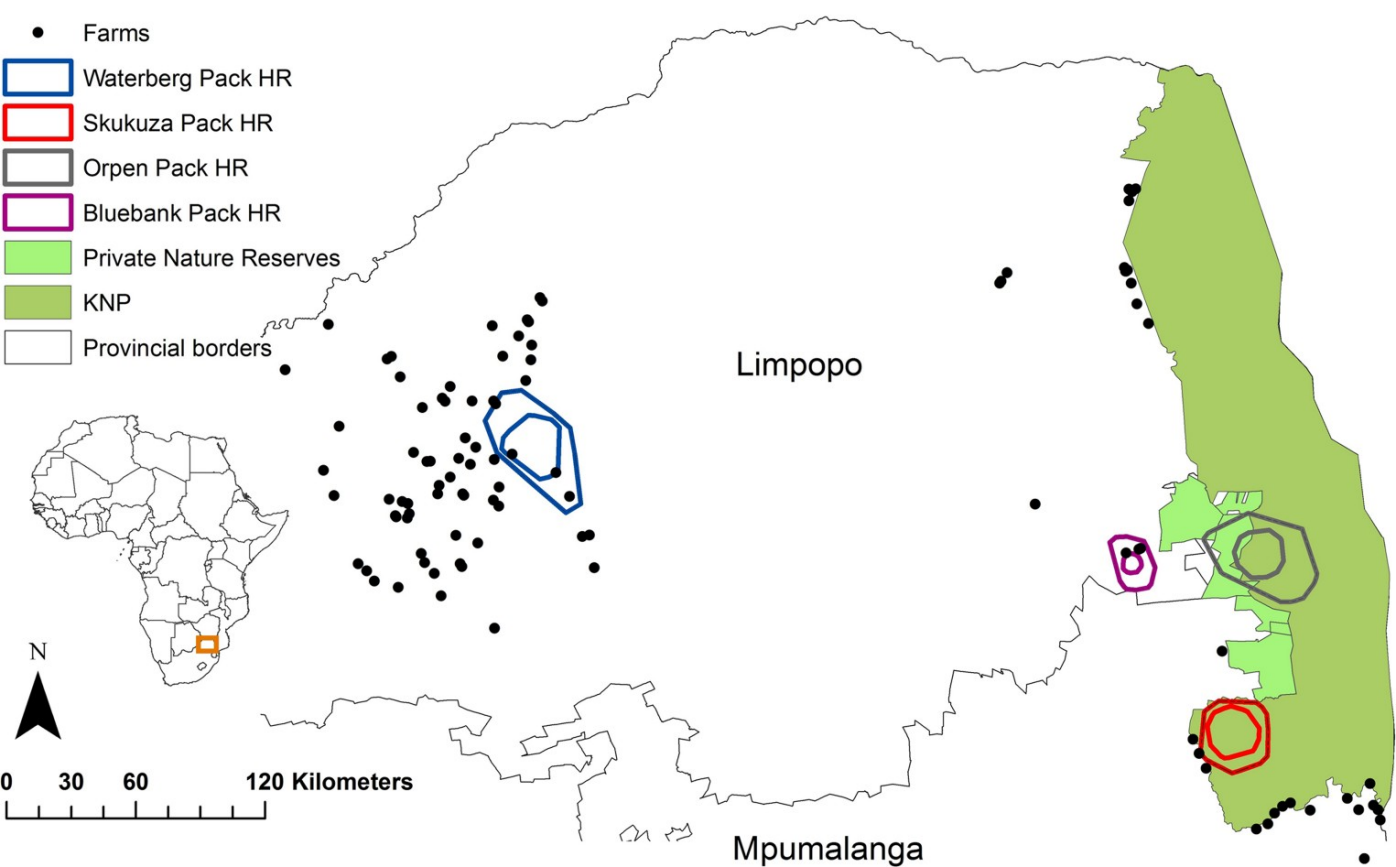
Home (95%) and core (50%) home ranges (HR) of four collared wild dogs, demarcated by coloured solid-line polygons, in relation to the location of farms (black dots) within the Limpopo and Mpumalanga provinces, South Africa. The Waterberg and Bluebank packs occurred outside protected areas, and the Skukuza and Orpen packs occurred within Kruger National Park (KNP). Farms consisted of a variety of subsistence and commercial farms (e.g. livestock, mixed-use or game ranching), all of which reported to practice lethal control of carnivores (Seoraj-Pillai 2016).

The four packs selected for collaring and use in our study occurred in the most northern part of South Africa, in the Limpopo and Mpumalanga provinces (Fig 1) and were radio-tracked from 2013 to 2015. Our sample size was limited by the availability of collars. Two packs occurred outside of national parks and ranged freely in the Waterberg area (named Waterberg and Bluebank) and two packs ranged within the western border of the KNP (named Skukuza and Orpen; Fig 1). The Waterberg biosphere was established to integrate biodiversity conservation with sustainable land management, including carnivore conflict mitigation, making this site ideal for investigating resource selection in free-ranging African wild dog [27]. Similarly, damage-causing carnivores have also been reported to originate from KNP, significantly affecting local communities on the park boundary [28].

Pack sizes were estimated as Waterberg (n = 7), Skukuza (n = 9), Orpen (n = 32) and Bluebank (n = 6), but it was difficult to verify pack sizes due to surrounding vegetation and elusiveness of individuals. A mixture of sour bushveld, thorn thickets and bushwillow *Combretum* spp. woodland predominates in the south-western border of the KNP, and Mopane *Colophospermum mopane* woodland, knob thorn *Acacia nigrescens* marula savannah and bushwillow woodland vegetation occurs on the western border of the KNP [29], where collaring occurred. The Waterberg is characterised by mountain and sandy bushveld types, with pockets of scrub and woodland [30]. The Bluebank area is typified by granite lowveld and sour bushveld vegetation [30]. These localities were also adjacent to abundant commercial and subsistence crop farms, including livestock and game pasturage with mixed farming practices [27]. The most notable agricultural activities in these regions include forestry, citrus *Rutacea*e spp. and tobacco *Nicotiana tabacum* farming, and cattle, goat *Capra aegagrus hircus* and poultry (Order: Galliformes) farming [31]. The area also contained game farms, involving the breeding and keeping of plains game [27]. The Limpopo and Mpumalanga provinces are also popular destinations for ecotourism, with a multitude of private game reserves as well as the KNP, a formally protected area [31]. Formally protected areas such as the KNP are clearly demarcated geographically and with a specific conservation aim and where no human interference activities (such as wood or animal harvesting) are allowed by law [32].

### Data collection

Data for this study were extracted from a single alpha individual per pack that was fitted with either a satellite or radio collar. For territorial, group-living canids, home ranges of individuals accurately reflect those of the group [33, 34], which is particularly appropriate for the cohesive pack structure of wild dogs. Research protocols and use of external radio transmitters followed the guidelines of the American Society of Mammalogists, with collars representing < 5% of wild dog body mass [35]. Two types of collars were used due to EWT specifications and funding constraints. Skukuza and Waterberg individuals were collared with global positioning system-ultra high frequency (GPS-UHF) collars, manufactured by Vectronic Aerospace. Iridium satellite wild dog collars (model G5C 275 D, manufactured by Sirtrack Ltd) were used to collar the Bluebank and Orpen individuals.

Collar sampling fixes were set at 4-hour intervals per day, which provides an adequate sample size for a species that can travel great distances daily [16] and avoids autocorrelation. Duplicate entries (fixes with identical date—time stamps) were removed. The movement of the collared alpha individuals was taken to represent the movement of the entire pack following a Design 2 pattern as outlined by Manly et al., p 6 [6]. In this design, individual animals were identified by radio-tracking collars and their use of resources, as obtained from classified aerial imagery, were measured for the individual animal, although resource availability could be extrapolated to the population level. To compare resource selection according to seasons, the availability data of the four packs were partitioned into the four seasons as obtained from the South African National Weather Service (Spring: 1 September—30 November, Summer: 1 December—28 February, Autumn: 1 March—31 May and Winter: 1 June—31 August). However, we obtained data for the four seasons for only the Bluebank pack due to the extended battery life of the Iridium collar. Data for the Orpen pack was collected in summer, autumn and winter, the Waterberg for spring, summer and autumn and the Skukuza pack for spring and summer. The Skukuza and Orpen packs were reported to have pups, but it was unclear whether the Waterberg and Bluebank packs were reproductively active during this study. Although wild dogs restrict their home range size during the denning period, these home ranges can still average 50–260 km^2^ [22], large enough to potentially bring wild dogs into contact with human—modified landscapes. We, therefore, did not distinguish between denning and non-denning periods in the study packs.

### Space use analyses

The space use patterns of the packs were analysed using Resource Selection Functions (RSFs) which are used to estimate the probability of a resource unit being used by a species [36]. The RSF method involves the comparison of the presence data of an animal at a resource (here derived from GPS collar fixes) to availability data, characterised as random points of resources accessible in the environment (see p 17) [6]. Following Beyer [37], we created Home Range Squares for each African wild dog pack in ArcMap (ArcGIS for desktop Version 10.1, Environmental Systems Research Institute (ESRI) Development Team), with each square encompassing all location fixes. These Home Range Squares were then imported into the Geospatial Modelling Environment (GME) software command builder (Version 0.7.2.RC2) to generate random points for each pack’s Home Range Square at a 1:1 ratio [37]. This random point generation method is a standard approach in RSF analysis because it requires presence/availability data or presence/absence data; true absence is almost impossible to quantify accurately since an organism might have been undetected and not truly absent [6]. Therefore, the generated random points were a measure of available resources for each African wild dog pack within its home range, whereas the original points represent true presence at the resource within the home range (see p 4) [6]. We selected anthropogenic explanatory variables that served as proxies for possible African wild dog mortality risk and used nature reserves as proxy areas of minimal human conflict for African wild dog resource selection (Table 1), which represented natural refuges with food and shelter [38]. Livestock density was included as a separate analysis [20]. Here, aside from livestock density, we reported only figures per feature name (e.g. Agriculture, Roads, Human Land use and Nature reserve) and season. Values for differences of distances of wild dog packs by feature subclass (Table 1) and Odds Ratios are provided in the supplementary tables (S1–S5 Tables).

**Table 1:** Description and characteristics of environmental features used in resource selection function analyses for four African wild dog packs in Limpopo and Mpumalanga provinces, South Africa. Feature subclasses and descriptions were taken from pre-existing shapefile attribute tables in ArcMap, except for agricultural landscape features, which we defined.

Feature name	Feature subclasses	Feature description
**Livestock density**	Cattle	Global livestock density per km^2^ downloaded and modified from the FAO. Sheep were omitted from this analysis, as the raster indicated that the study sites contained zero (low) densities of sheep, with high densities occurring in the drier, south-western parts of South Africa
	Goats	
	Poultry	
**Agriculture**	General farm	Farms keeping multiple livestock types, game or both (mixed purpose)
	Hunting lodge	Lodge where plains game are kept only for the purpose of trophy/meat hunting
	Game farm	Farms where plains game are bred for sale/conservation purposes
	Poultry farm	Farms producing exclusively poultry products (meat/eggs/feathers)
	Goat farm	Farms producing exclusively goat products (meat/milk/cheese/mohair)
	Fruit and Nut	Farms producing exclusively fruit and nut crops
**Roads**	Motorway	Highway, speed limit 120 km/h
	Motorway link	Connecting road for highways, speed limit 120 km/h
	Trunk	Major road, speed limit 120 km/h
	Trunk link	Connecting road for major roads, speed limit 80 km/h
	Primary	Public road outside urban area, speed limit: 100 km/h
	Secondary	Public road within a nature reserve, speed limit 50 km/h
	Residential	Public road within an urban area, speed limit 60 km/h
	Tertiary	Dirt road, speed limit: 30 km/h
	Track	Secondary rough dirt road, speed limit 20km/h
	Unknown	Unclassified road, speed limit 20km/h
	Footway	Trails used by humans to travel on foot
**Human land use**	Residential	Housing predominated area
	Commercial	Area predominated by businesses and office complexes
	Reservoir	Artificial storage area for water
	Quarry	Excavation site for rock and other construction aggregate
	Cemetery	Burial place of human remains
	Recreational	Grassy area used for sports and other outdoor activities
	Retail	Area predominated by shopping centres
	Industrial	Heavily urbanised area with many factories
	Military	Areas occupied by the Department of Defence
	Landfill	Site for the disposal of waste materials
	Railway	Permanent track for transportation by train
**Nature reserve**	National	State owned or privately run areas under formal protection for the conservation of wildlife and ecosystems
	Private	

We quantified the distance to the presence and random location points for the subclasses of nature reserves, agricultural landscape features (such as farms), roads, and other human land uses (in km) or intersect with livestock density (number of animals per km^2^). Due to zero raster values in areas of pack occurrence, sheep *Ovis aries* were omitted from the analyses. Agricultural landscape feature data (Table 1) were obtained from a previous study by Seoraj-Pillai [39]. We assumed the African wild dogs would occur at a feature if the median distances of presence data to the features were < 10 km, since studies have shown that African wild dogs travel average distances of 8.5–10.5 kilometres daily within their territories, with minimum travel distances of 5–6 kilometres daily [40, 41]. Because the Skukuza and Orpen packs occurred within a nature reserve (KNP), the occurrence distance to this particular reserve was 0 km, and other distances recorded reflected other nature reserves potentially available for these packs.

### Statistical analyses

We used RStudio Desktop software (R version 3.5.1; http://www.R-project.org) for all statistical analyses. To determine which of the five variables (livestock density, agricultural landscape features, roads, human land use and nature reserves) best predicted space use patterns for African wild dog per season, we performed a multi-model selection using the Akaike Information Criterion (AIC) and delta Akaike (ΔAIC) (MuMIn package) [42]. General linear models were then constructed to test the best candidate model, seasonal effects and feature type space use per pack (glmmsr package) [43], using those models with the lowest ΔAIC values. The African wild dog packs were not sampled equally across all seasons and experienced different landscape features in protected and unprotected areas. Therefore, we did not make statistical comparisons between packs but separately analysed the data for each pack. Livestock density and distance to agricultural landscape features, roads, human land use and nature reserves were continuous variables, and the availability of the features versus the actual presence of African wild dogs at these features was a binomial variable. Odds Ratios (ORs) were calculated for all feature subclasses per wild dog pack to assess the probability of wild dog occurrence in relation to anthropogenic and natural landscape features. Odds Ratios are used for binary data as measures of association when comparing exposure and outcome [44]. ORs represent the chance that a specific exposure will lead to an outcome, versus the chance of an absence of that exposure creating an outcome [44]. When OR = 1.0, the chance of either outcome occurring is equally likely [45]. When OR < 1, the chance of the outcome occurring is less likely and when OR > 1 the chance of the outcome occurring is more likely [45]. Hence for our study, if OR = 1.0, the odds of occurrence or non-occurrence of the wild dog pack at a feature (either natural or anthropogenic) was equal. When OR < 1, the odds of occurrence of a wild dog pack at a feature was low, and if OR > 1, the odds of occurrence at a feature was high. It must be noted that, as mentioned previously, non-occurrence may not mean absence, as the animal may have been undetected and not truly absent [6].

## Results

The Bluebank pack was tracked in spring summer, autumn and winter (n = 1456 locality fixes, 389 days monitored), the Waterberg pack in spring, summer and autumn (n = 399, 110 days monitored), the Orpen pack in summer, autumn and winter (n = 492, 150 days monitored) and the Skukuza pack in spring and summer (n = 304, 79 days monitored). Of the five landscape features tested in relation to each of the African wild dog packs, livestock density, agricultural features, roads, human land use and nature reserves were the best predictors of space use for the Waterberg and Bluebank packs (Table 2). As expected, livestock density was not a predictor of space use for either the Skukuza or Orpen packs (Table 2). Additionally, human land use was not a predictor of space use for the Orpen pack (Table 2). Results below are described per pack according to AIC outcomes.

**Table 2:** Akaike Information Criterion (AIC) regression models investigating the effects of different features in a landscape on the space use of four African wild dog packs in the Limpopo and Mpumalanga provinces, South Africa. Columns marked with an ‘X’ indicate features included in the models. The most parsimonious models are indicated in bold for each pack and were used in all subsequent analyses.

Pack	Livestock density (km^2^)	Agricultural features (km)	Roads (km)	Human Land use (km)	Nature Reserve (km)	df	AIC	ΔAIC	Weight
Waterberg	**X**	**X**	**X**	**X**	**X**	**7**	**1022.6**	**0.00**	**0.417**
		X	X	X	X	6	1024.5	1.96	0.157
	X	X		X	X	6	1025.2	2.59	0.114
Skukuza		**X**	**X**	**X**	**X**	**9**	**1831.2**	**0.00**	**0.558**
			X	X	X	8	1831.7	0.47	0.442
		X	X		X	8	1849.0	17.75	0.000
Orpen		**X**	**X**		**X**	**8**	**-1872.8**	**0.00**	**0.677**
		X	X		X	7	-1871.3	1.48	0.323
		X			X	7	-1688.7	184.11	0.000
Bluebank	**X**	**X**	**X**	**X**	**X**	**9**	**1831.2**	**0.00**	**0.389**
		X	X	X		8	1831.7	0.47	0.308
	X	X	X	X	X	10	1832.9	1.62	0.173

### Livestock density

**Fig 2:**
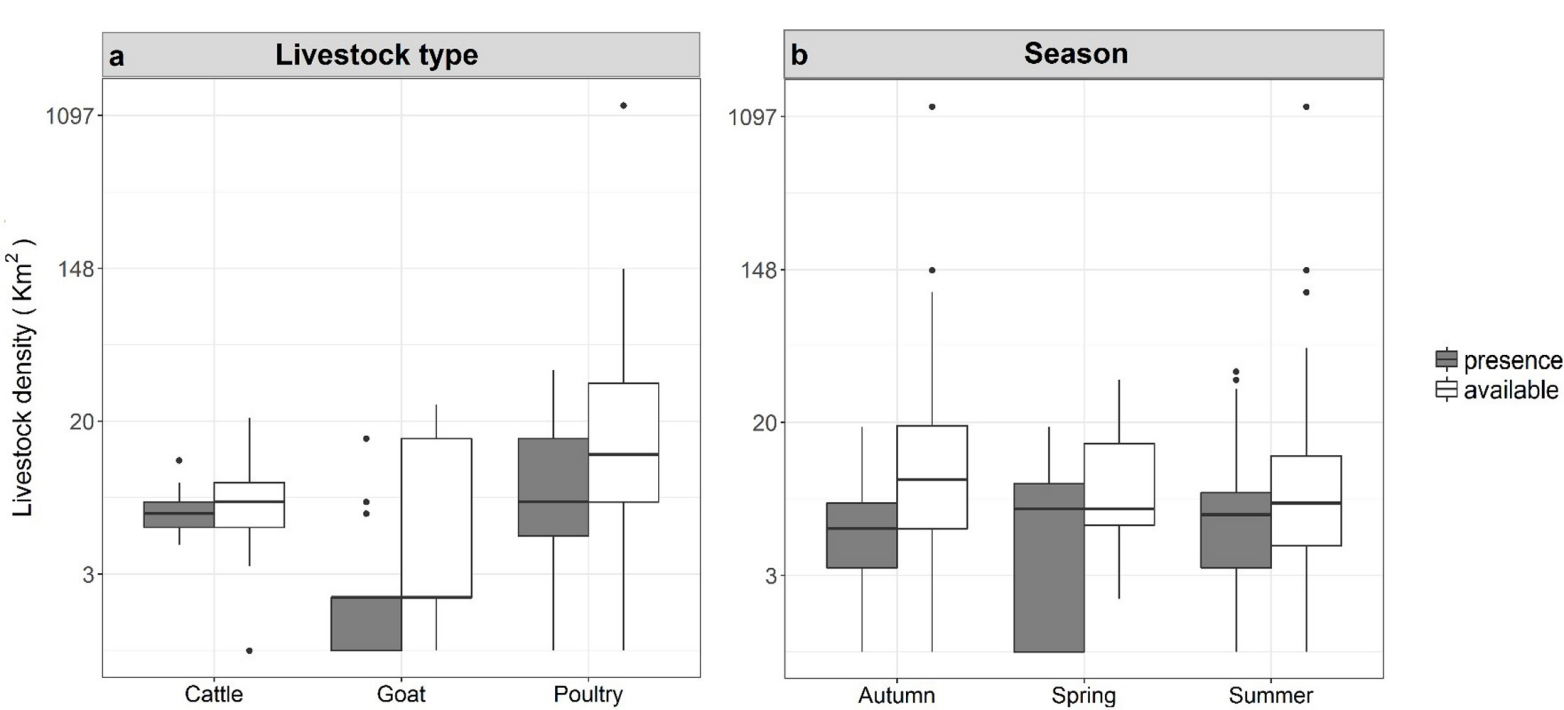
**The presence and availability data for the Waterberg wild dog pack in a) areas with different livestock type (cattle, goats and poultry) densities (per km2) and presence and availability data across b) different seasons in Limpopo Province, South Africa.** Boxplots show medians (dark horizontal bars), 1st and 3rd interquartiles (boxes), 95% CI (whiskers) and outliers (dots). Presence data are indicated by coloured boxes.

**Fig 3:**
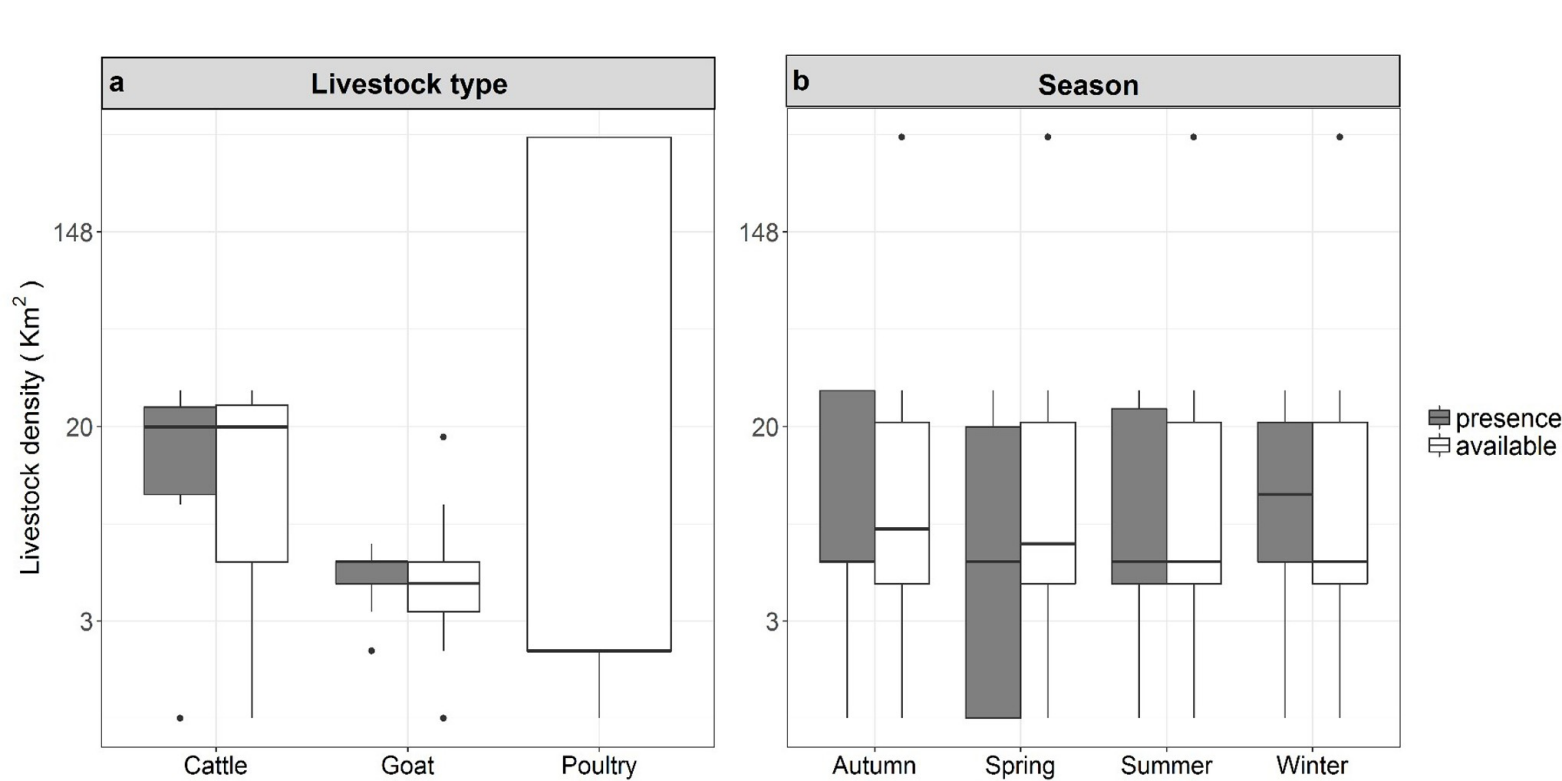
**The presence and availability data for the Bluebank wild dog pack in a) areas with different livestock type (cattle, goats and poultry) densities (per km2) and presence and availability data across b) different seasons in the Mpumalanga Province, South Africa.** Boxplots show medians (dark horizontal bars), 1st and 3rd interquartiles (boxes), 95% CI (whiskers) and outliers (dots). Presence data are indicated by coloured boxes.

The Waterberg pack occurred in areas of lower livestock density (km^2^) than were available in the environment (χ^2^_1_ = 132.25, p < 0.001; Fig 2A). There was no difference between Waterberg pack presence and the availability for livestock densities during different seasons (χ^2^_2_ = 2.58, p = 0.275; Fig 2B). The Waterberg pack presence was greater near areas of higher poultry densities than goat or cattle densities (χ^2^_2_ = 702.92, p < 0.001). This pack’s odds of occurrence were high for areas that housed cattle, and lower for areas that housed poultry (S1 Table). The odds of occurrence were low in areas that contained goats (S1 Table). The Bluebank pack occurred in areas of lower livestock density (km^2^) than were available in the environment (χ^2^_1_ = 13.64, p < 0.001; Fig 3A). There was a significant influence of season (χ^2^_3_ = 8.17, p = 0.042; Fig 3B), with the Bluebank pack occurring in areas of higher livestock densities in autumn and winter than were available in the environment. The Bluebank pack occurred in areas of higher cattle than goat densities but did not occur in areas of containing poultry (χ^2^_2_ = 356.49, p < 0.001). Odds of occurrence of the Bluebank pack in areas of different livestock type densities were highest in areas that contained cattle (S1 Table). Odds of occurrence were lower in areas that contained poultry, and lowest in areas that contained goats (S1 Table).

### Agricultural landscape features

**Fig 4:**
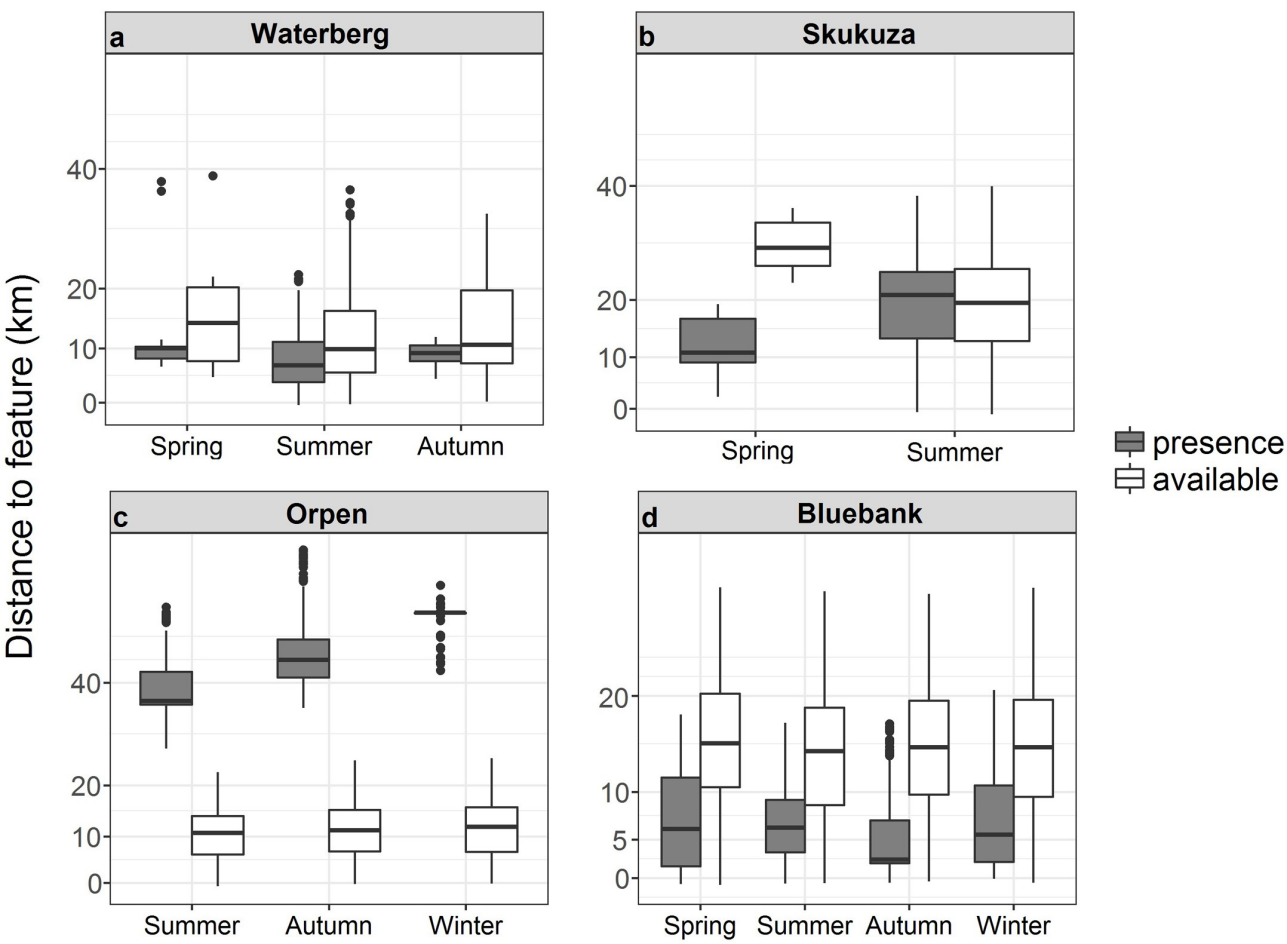
**African wild dog pack presence and availability data in areas of agricultural landscape feature types for the a) Waterberg, b) Skukuza, c) Orpen and d) Bluebank packs across different seasons in the Limpopo and Mpumalanga Provinces, South Africa.** Boxplots show medians (dark horizontal bars), 1^st^ and 3^rd^ interquartiles (boxes), 95% CI (whiskers) and outliers (dots). Presence data are indicated by coloured boxes.

The Waterberg pack occurred significantly closer to agricultural landscape features than were available in the environment (χ^2^_1_ = 7.9.21, p < 0.001), and the pack occurred closer to agricultural features in summer than spring or autumn (χ^2^_2_ = 95.98, p = 0.009; Fig 4A). The Waterberg pack occurred in closer proximity to general farms than hunting lodges (χ^2^_2_ = 335.14, p < 0.001; S2 Table). The pack’s odds of occurrence were greatest for general farms, with equal odds of occurrence or non-occurrence at hunting lodges and low odds of occurrence at goat farms (S2 Table). The Skukuza pack occurred in different proximities to agricultural landscape features than were available in the environment (χ^2^_1_ = 7.03, p < 0.001). The Skukuza pack presence at agricultural landscape features differed during the spring and summer (χ^2^_1_ = 134, p < 0.001; Fig 4B), occurring further away from features during summer than spring (Fig 4B). The Skukuza pack occurred further away from the poultry farm than the general farms (χ^2^_11_ = 158.71, p < 0.001), with the pack occurring >10 km from general farms and > 20 km from poultry farms (S2 Table). The Skukuza pack’s odds of occurrence were greatest at agricultural landscape features for poultry and game farms and lower for fruit and nut farms and general farms (S2 Table). The Orpen pack occurred significantly further away from agricultural landscape features than were available in the environment (χ^2^_1_ = 1720.06, p < 0.001). Availability data for the Orpen pack indicated possible occurrence at distances > 10 km and presence data indicated occurrence at distances > 40 km (Fig 4C). The pack’s presence differed at agricultural landscape features during seasons (χ^2^_2_ = 49.97, p < 0.001; Fig 4C), occurring further away during winter (> 40 km) and closer during summer (> 20 km). Similarly, the pack occurred significantly further away from poultry farms than fruit farms (χ^2^_3_ = 56.36, p < 0.001), but with odds of occurrence indicating a high likelihood of occurrence at poultry farms and a low occurrence at fruit farms (S2 Table). The Bluebank pack occurred in closer proximity to agricultural features when compared to availability data (χ^2^_1_ = 1485.29, p < 0.001). The Bluebank pack occurrence was closer to farms for all seasons compared to availability data (χ^2^_3_ = 100.73, p < 0.001), especially in autumn (Fig 4D). Presence at agricultural features differed (χ^2^_3_ = 100.73, p < 0.001) with no pack occurrence close to game farms, although odds of occurrence were high for game farms (S2 Table).

### Roads

**Fig 5:**
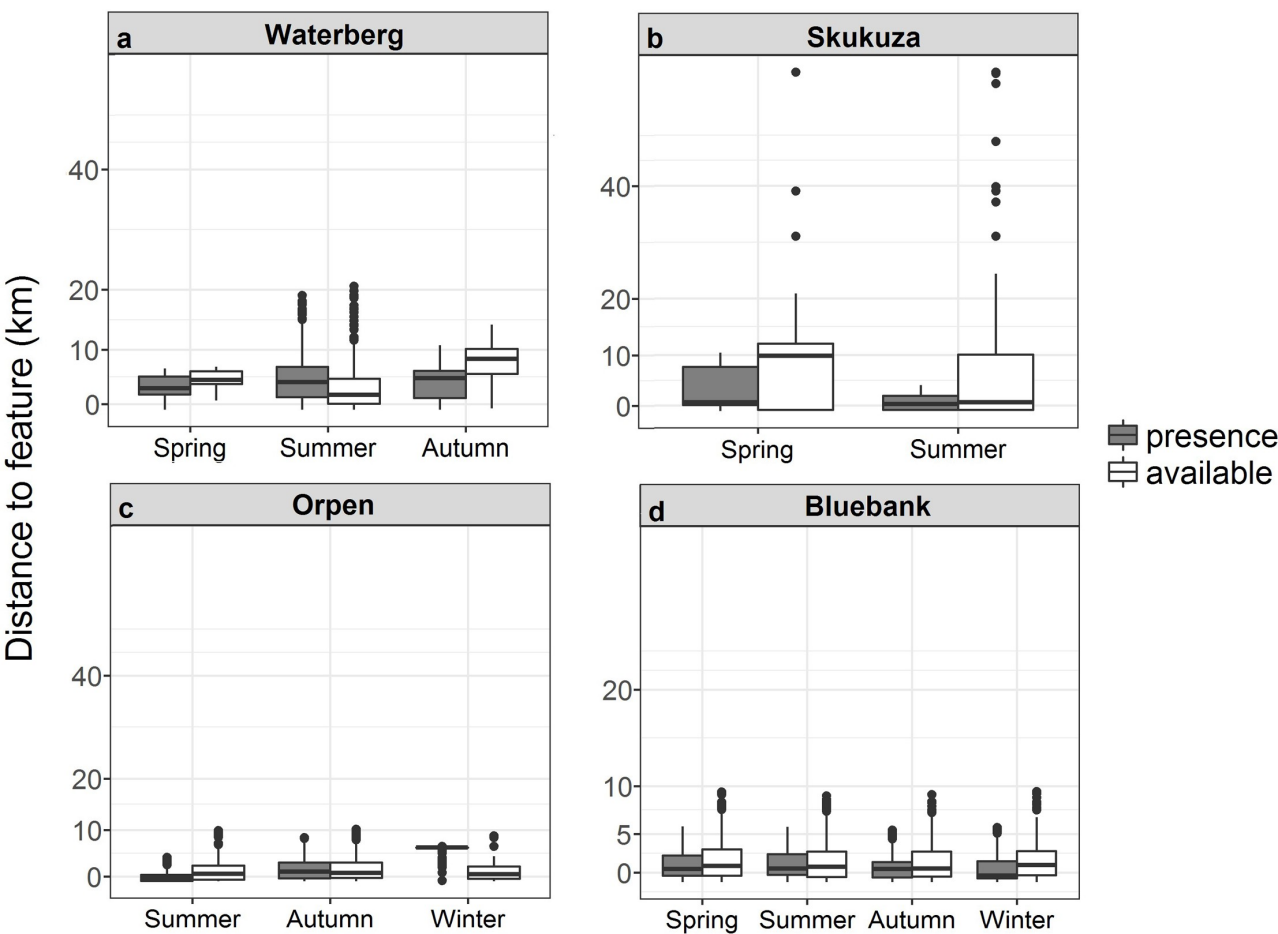
**African wild dog pack presence and availability data in areas of roads for the a) Waterberg, b) Skukuza, c) Orpen and d) Bluebank packs across different seasons in the Limpopo and Mpumalanga Provinces, South Africa.** Boxplots show medians (dark horizontal bars), 1^st^ and 3^rd^ interquartiles (boxes), 95% CI (whiskers) and outliers (dots). Presence data are indicated by coloured boxes.

The Waterberg pack occurred further from roads than were available in the environment (χ^2^_1_ = 55.58, p < 0.001). The Waterberg pack occurred closer to roads during autumn (χ^2^_2_ = 30.07, p <0.001; Fig 5A). There were no differences between the Waterberg pack’s presence at different road types or availability of these road types in the environment (χ^2^_9_ = 9.84, p = 0.363), with occurrences at > 9 km (S3 Table). The Waterberg pack’s odds of occurrence on motorways and motorway links (both high-speed roads) were high, but were low on primary, trunk and trunk-link roads (S3 Table). The odds of occurrence on residential roads (moderate-speed roads) were high, but odds of occurrence on secondary roads were low and other roads carrying slow-moving traffic (tertiary, track and unclassified) were low (S3 Table). The Skukuza pack occurred significantly closer to roads within Kruger National Park than available in the environment (χ^2^_1_ = 47.03, p <0.001). The Skukuza pack also occurred at different distances to roads during different seasons (χ^2^_1_ = 8.60 p = 0.003; Fig 5B), occurring closer to roads during summer than spring. The pack occurred at different distances to different road types (χ^2^_4_ = 36.51 p < 0.001), occurring jointly closer to secondary and unclassified roads (> 1 km) than tertiary or track roads (S3 Table). The pack’s odds of occurrence on roads that carry slow-moving traffic (tertiary and track) were high, but odds of occurrence or non-occurrence on unclassified roads were equal (S3 Table). The odds of occurrence on moderate speed roads (secondary) were low (S3 Table).

There was no difference between availability and presence data on roads for the Orpen pack (χ^2^_1_ = 0.33 p = 0.566), but this pack showed a trend for the types of roads utilised (χ^2^_9_ = 16.04, p = 0.066). The Orpen pack occurred at roads at different distances during different seasons (χ^2^_2_ = 183.42, p < 0.001), being closer to roads in summer than autumn or winter (Fig 5C).

The Orpen wild dog pack’s odds of occurrence were high on all roads carrying fast-moving traffic (motorway, motorway link, primary and trunk link), except for trunk roads (S3 Table). Odds of occurrence for moderate-speed roads were high on residential road types but were low secondary road types (S3 Table). Pack occurrence on slow-moving road types was low for tertiary and track roads but high for unclassified roads (S3 Table). The Bluebank pack occurred significantly different distances to roads during different seasons (χ^2^_3_ = 14.89, p = 0.001; Fig 5D), occurring closer to roads during winter and autumn than spring or summer. The pack did not occur closer to certain road types than others (χ^2^_10_ = 16.61, p = 0.083), with odds of occurrence being equally likely for the motorway road type, but were high for all other high-speed road types (motorway link, primary and trunk) (S3 Table). Odds of occurrence were high on moderate-speed road types (residential and secondary) and all slow-speed road types (path, tertiary, track and unclassified), except for footway road types (S3 Table).

### Human land use

**Fig 6:**
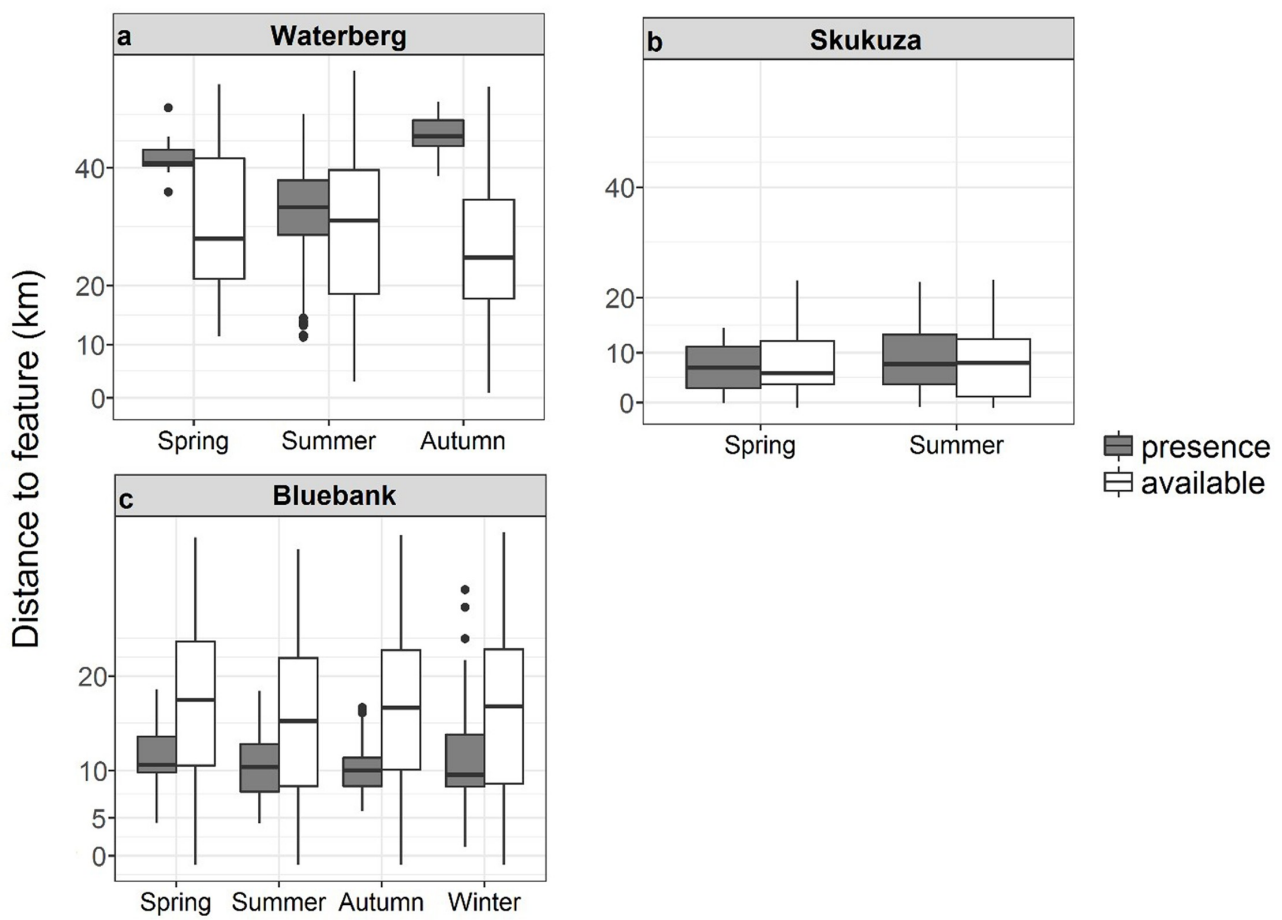
**African wild dog pack presence and** availability **data in areas of different human land use feature types for the a) Waterberg, b) Skukuza and c) Bluebank packs across different seasons in the Limpopo and Mpumalanga Provinces, South Africa.** Boxplots show medians (dark horizontal bars), 1^st^ and 3^rd^ interquartiles (boxes), 95% CI (whiskers) and outliers (dots). Presence data are indicated by coloured boxes.

The Waterberg pack occurred significantly further away from human land use landscape features than were available in the environment (χ^2^_1_ = 43.93, p < 0.001). The Waterberg pack was further away from these features during autumn than spring and summer (χ^2^_2_ = 20.40, p < 0.001; Fig 6A). There was no difference between occurrence at different types of human land use features (χ^2^_14_ = 21.87, p = 0.081). The Waterberg pack’s odds of occurrence or non-occurrence at human land use types were equal for industrial sites, and almost equal for cemetery, commercial, recreational and reservoir sites (S4 Table). Odds of occurrence were high for landfill and residential sites and low for military, quarry, railway and retail sites (S4 Table). The Skukuza pack occurred significantly different distances away from human land use features that were available in the environment (χ^2^_1_ = 14.79, p < 0.001). Distance to human land use did not show seasonal differences for the Skukuza pack (χ^2^_1_ = 1.17, p = 0.278; Fig 6B). No differences existed between pack proximity to the different types of human land use features (χ^2^_14_ = 18.57, p = 0.182). The Skukuza pack’s odds of occurrence at human land use types were almost equal for railway sites (S4 Table). Odds of occurrence were high for commercial, industrial, military, quarry, recreational, reservoir and retail sites (S4 Table). Odds of occurrence were low for cemetery, landfill and residential sites (S4 Table). The Bluebank pack occurred significantly closer to human landscape features than were available in the environment (χ^2^_1_ = 401.42, p < 0.001). Occurrence of the Bluebank pack at landscape features differed during seasons (χ^2^_3_ = 17.89, p < 0.001; Fig 6C), occurring further away in spring than autumn. This pack occurred close to military sites but not reservoirs (χ^2^_1_ = 97.10, p < 0.001), although odds of occurrence were high at reservoirs and low at military sites (S4 Table).

### Nature reserves

**Fig 7:**
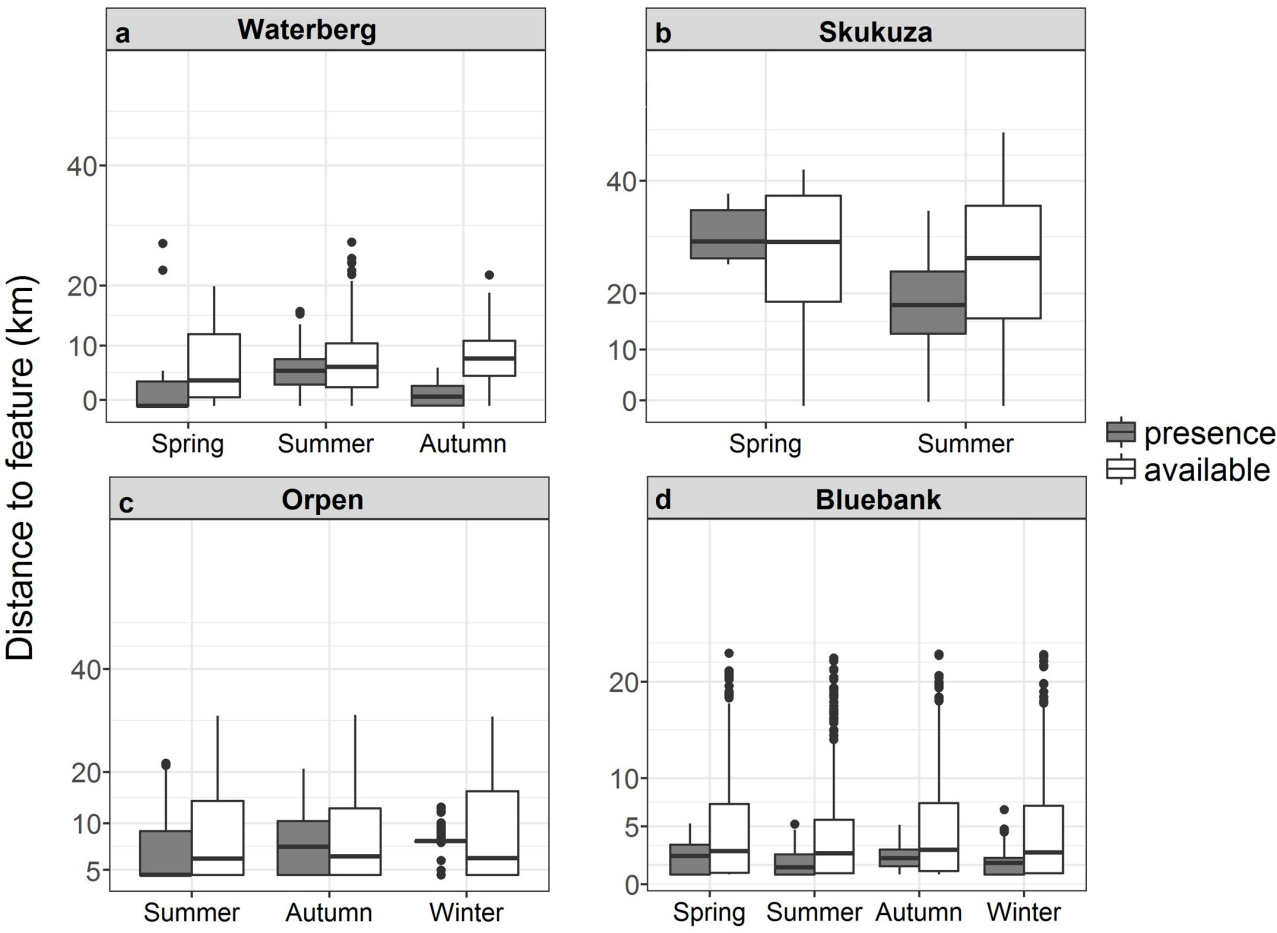
**African wild dog pack presence and availability data in areas of nature reserves for the a) Waterberg, b) Skukuza c) Orpen and d) Bluebank packs across different seasons in the Limpopo and Mpumalanga Provinces, South Africa.** Boxplots show medians (dark horizontal bars), 1^st^ and 3^rd^ interquartiles (boxes), 95% CI (whiskers) and outliers (dots). Presence data are indicated by coloured boxes.

The Waterberg pack occurred closer to nature reserves than were available in the environment (χ^2^_1_ = 10.90, p < 0.001). The Waterberg pack occurred at nature reserves differently during different seasons (χ^2^_2_ = 22.13, p < 0.001; Fig 7A), occurring closer to reserves in autumn and spring than summer. Similarly, this pack utilised different nature reserves (χ^2^_13_ = 286.73, p < 0.001; S5 Table), The Skukuza pack occurred closer to nature reserves situated outside KNP than was available in the environment (χ^2^_1_ = 21.25, p < 0.001). Their occurrence differed seasonally (χ^2^_1_ = 16.62, p < 0.001; Fig 7B), with the pack occurring closer to reserves during summer than spring. The Orpen pack occurred further away from nature reserves outside of KNP than was available (χ^2^_1_ = 2058.90, p < 0.001). The Orpen pack occurrence differed seasonally (χ^2^_2_ = 41.40, p < 0.001; Fig 7C), and was closer to reserves during winter and further away in summer. This pack occurred at different distances to different reserves (χ^2^_6_ = 217.99, p < 0.001shown by a 0.00 median distance, S5 Table). The Bluebank pack occurred significantly closer to nature reserves than available in the environment (χ^2^_1_ = 72.58, p < 0.001). Bluebank pack occurrence at reserves differed during seasons (χ^2^_3_ = 20.98, p < 0.001; Fig 7D), occurring closer to reserves during summer than autumn, spring or winter. The pack occurred at different distances to different reserves (χ^2^_12_ = 1236.39, p < 0.001; S5 Table)

## Discussion

Along with a variety of other factors responsible for the population decline of the endangered African wild dog, one of the most notable threats is the conflict with humans. We considered four packs in our study, two of which occurred outside of formally protected nature reserves, and two packs occurred in the Skukuza and Orpen areas of the Kruger National Park (KNP), together comprising approximately 10% of the total African wild dog population in South Africa. Although packs outside of KNP were located in areas of high cattle and goat densities and could access these areas, their actual occurrence was more often in areas housing lower livestock densities per km^2^. African wild dog may pre-emptively avoid (i.e. predictive avoidance) farmland due to previous negative experiences, such as observing mortality of pack members [7, 8]. Predictive avoidance behaviours have been shown in lion *Panthera leo* avoiding cattle posts in Botswana [8] puma *Puma concolor* avoiding areas of high human activity, such as housing neighbourhoods, in California [46] and brown bear *Ursus arctos* avoiding areas of human habitation during the daytime in Scandinavia [47].

Additionally, African wild dog may be naturally cautious of farmlands due to their innate fear of humans [48, 49]. The Bluebank pack showed seasonal differences in relation to livestock density, occurring in areas of higher livestock density during autumn and winter. Such changes in occurrence might have occurred for a number of undetected reasons that we hypothesise are related to breeding. African wild dog breed during the autumn and winter months (April—June) when resource requirements of the pack increase [50]. Most wild herbivores give birth during the summer and spring months when forage is abundant [51] whereas livestock may have variable breeding seasons. Beef cattle have distinct calving times in summer from January to February [52], but Boerbok goats *Capra aegagrus hircus* breed are able to produce young at any time of the year, showing peaks in reproduction during the autumn months [53]. Boerbok is common livestock throughout South Africa due to their versatility, hardiness and ease of keeping [53]. The presence of livestock young (e.g. Boerbok kids produced in autumn) might have attracted the pack during their breeding period.

Depredation of livestock might, therefore, be isolated incidents. The African wild dog diet is flexible with a broad dietary niche [54], easily switching from smaller-bodied prey such as oribi *Ourebia ourebi* to their preferred prey impala *Aepyceros melampus*, or large-bodied prey such as waterbuck *Kobus ellipsiprymnus* or kudu *Tragelaphus strepsiceros* [55]. It also prefers natural prey, even when free-roaming livestock is available [21, 40, 56] but will switch to livestock when natural prey is severely depleted [57]. Unconfirmed reports obtained from interview data of farmers in the Waterberg suggest that a mixture of livestock and game, including wild ungulates, have been killed by wild dogs [39]. This suggests that wild dogs can depredate a variety of wild and farmed species. Areas outside of protected areas sometimes contain less natural prey due to anthropogenic influences and hunting [58].

The two wild dog packs inside the KNP occurred at distances exceeding 100 km to agricultural features. The two packs outside of KNP occurred closer to agricultural features than available in the environment, although the median distance of occurrence was not less than 40 km. Kruger National Park conservation and veterinary authorities monitor wildlife permeability and damage to fences along the western boundary fence of the KNP [59]. The western perimeter fence differs in strength and structure to manage or buffer different intensities and sources of damage [60]. The KNP perimeter fences have become permeable due to flooding and the fence-pushing behaviour of bull elephants *Loxodonta africana* [59]. In addition, older non-functional electric fences on the western border have become the most permeable to wildlife, especially to elephants and large carnivores [59]. Due to the wide-ranging behaviour of African wild dog [16] and their long-distance movements within the KNP [40], as well as gaps in the border fences [59], it is likely that African wild dog individuals may frequently use unprotected areas adjacent to the KNP [61]. However, recent research by Parker et al. [62] indicates that the African wild dog packs on the western boundary of the KNP are buffered by contiguous surrounding nature reserves (e.g. the APNR). This together with the positive attitudes to the species by surrounding citrus farmers will provide a measure of protection for African wild dog regionally.

Roads were an important predictor of African wild dog occurrence and therefore a potential risk. Due to their occurrence outside of the KNP, the Waterberg and Bluebank packs occurred in close proximity to major roads (tarred multi-lane roads with speed limits of 120 km/h) at distances < 20 km. The two packs occurring within the KNP, particularly the Orpen pack, occurred very close to roads (tarred and dirt), with presence data indicating occurrence distances of < 10 km. All three packs had a high likelihood of encountering major roads that carry fast-moving vehicular traffic Within KNP, strict maximum speed limits of 50 km/h apply on tarred roads and 40 km/h on dirt roads [63], and therefore these roads might do not pose a mortality risk to wild dogs. While roads within reserves enhance landscape permeability to aid wild dog travel and space use [25] and are often used for herbivore flushing and capture [64], major roads outside of reserves and protected areas carry traffic moving at high speeds and pose a serious mortality risk due to vehicular collisions [65]. As human-altered landscapes are increasingly expanding, animals encounter major roads more often [66] and automobile collisions are considered as a leading source of human-caused vertebrate mortality [67]. Wild dogs are often accidentally killed when attempting to cross major roads [17], but they may also be in danger of being deliberately struck by motorists [68].

The wild dog packs showed seasonal differences in proximity to roads, with the Waterberg, Skukuza and Orpen packs occurring closer to roads in summer, and the Bluebank pack occurring closer to roads during autumn and winter. Life history events, such as dispersal or breeding, can affect space use and therefore the probability of animals encountering roads [69]. Animals may need to range far in search of food [69]. For example, in southern Portugal, red fox *Vulpes vulpes* and stone marten *Martes foina* encounter roads more during the breeding season when the risk of road mortality increases during this period [70]. In addition, herbivores may use roads in different seasons when moving to different foraging patches [71], attracting carnivores. For example, caribou *Rangifer tarandus* in Alaska use roads in winter when travelling to find new feeding sites, resulting in increased encounters with and mortality by grey wolves *Canis lupus* [72].

Human landscape features were not predictors of African wild dog occurrence for three packs; the Orpen pack was excluded from the analysis since they were not observed to move outside the KNP boundary. The Waterberg pack occurred further away from human land use features than were available in the environment and occurred at distances greater than 200 km. While the Bluebank pack occurred closer to human land use features than available in the environment, actual presence distances of the pack to land use features were not less than 100 km. We considered human land use as commercial properties, industrial properties (factories), quarries, mines, retail properties and railway lines [73]. These areas are highly urbanised and hubs of noisy human activity [73] and because wild dogs are generally wary of humans and their [48], they would avoid these areas. The Skukuza pack occurred close to the large commercial rest camp Skukuza (4.98 km^2^) in KNP, which we considered as human land use. Most of the major camps in KNP are built close to natural or artificial water sources [74], attracting many different herbivore species. For example, the Skukuza rest camp is built on the banks of the large, perennial Sabie River. Wild dogs have been documented to use camp and boundary fences as a tool to aid in prey capture, especially larger prey such as kudu and waterbuck [20, 75, 76]. Moreover, wild dogs habituate to human presence at campsites in the KNP [77], potentially bringing them into contact with similarly habituated herbivore prey.

Nature reserves were of particular importance to the Waterberg and Bluebank packs. Both packs occurred at distances < 10 km to nature reserves and showed high odds of occurrence within the surrounding reserves, which these packs might utilise. The Orpen pack also occurred < 10 km to reserves other than the KNP which is expected since the landscape is interspersed with nature reserves. Notably, the Timbavati private nature reserve adjacent to the KNP, sharing an unfenced boundary [59], and may be an important resource area for this pack. Although the Skukuza pack remained within KNP, the Sabie Sands private nature reserve would be easily accessible for the pack, since it occurs adjacent to the KNP, separated by only by the Sabie River and an unfenced boundary [59]. Protected areas are important for wide-ranging or migratory animals because they provide valuable resources, such as shelter and food [78]. Ecologically, protected areas are important for the establishment of meta-populations, and movement of individuals between meta-populations would ensure gene flow and species persistence [79]. Outbreeding is particularly critical for endangered species such as the African wild dog because their small population sizes make particularly prone to Allee effects and the resultant problems, such as disease outbreaks [80].

## Conclusions

Our research focussed on the association of the critically endangered African wild dog with anthropogenic features that might lead to their demise. We showed that the two packs outside protected reserves had access to many resources in the environment, including areas close to farms with livestock. Yet, as we predicted, these canids used areas with few farms and low livestock densities. This indicates that African wild dogs avoid farms and areas of high livestock densities. Nonetheless, the packs outside the KNP might be vulnerable to attacks by farmers, who report conflict with African wild dogs [81]. Contrary to expectations, these packs occurred near major roads, which is of particular concern because of the risk from automobile traffic. Future studies should focus on the vulnerabilities of packs outside of protected areas by cross-referencing wild dog presence on farmlands, roads and near human landscape features with reported livestock/ game mortalities as well as wild dog behaviour at these different sites. The extrapolations and conclusions made here are based on, and limited to, results from analyses conducted on our collared individuals in geographically distant locations. Studying a greater number of packs over a range of geographical areas are necessary to assess the generalisability of our findings.

## Supporting information

S1 Table Two African wild dog packs’ distances to different livestock densities outside of protected areas in the Limpopo and Mpumalanga provinces, South Africa, by availability and presence data, number of location points per feature subclass (n), feature subclass, median distance (km), confidence interval (CI) and odds ratio. Odds ratios (ORs) were calculated as the difference between availability data and presence data (thus presence ORs = n/a) and indicate the probability of occurrence of a wild dog pack at any given agricultural feature subclass. OR = 1 indicates equal chance of occurrence, OR < 1 indicates low chance of occurrence and OR > 1 indicates high chance of occurrence.
(DOCX)

S2 Table Four African wild dog packs’ distances to different agricultural landscape features in the Limpopo and Mpumalanga provinces, South Africa, by availability and presence data, number of location points per feature subclass (n), feature subclass, median distance (km), confidence interval (CI) and odds ratio. Odds ratios (ORs) were calculated as the difference between availability data and presence data (thus presence ORs = n/a) and indicate the probability of occurrence of a wild dog pack at any given agricultural feature subclass. OR = 1 indicates equal chance of occurrence, OR < 1 indicates low chance of occurrence and OR > 1 indicates high chance of occurrence.
(DOCX)

S3 Table Four African wild dog packs’ distances to different road types in the Limpopo and Mpumalanga provinces, South Africa, by available and presence data, number of location points per feature subclass (n), feature subclass, median distance (km), confidence interval (CI) and odds ratio. Odds ratios (ORs) were calculated as the difference between availability data and presence data (thus presence ORs = n/a) and indicate the probability of occurrence of a wild dog pack at any given agricultural feature subclass. OR = 1 indicates equal chance of occurrence, OR < 1 indicates low chance of occurrence and OR > 1 indicates high chance of occurrence.
(DOCX)

S4 Table Four African wild dog packs’ distances to different land use types in the Limpopo and Mpumalanga provinces, South Africa, by availability and presence data, number of location points per feature subclass (n), feature subclass, median distance (km), confidence interval (CI) and odds ratio. Odds ratios (ORs) were calculated as the difference between availability data and presence data (thus presence ORs = n/a) and indicate the probability of occurrence of a wild dog pack at any given agricultural feature subclass. OR = 1 indicates equal chance of occurrence, OR < 1 indicates low chance of occurrence and OR > 1 indicates high chance of occurrence.
(DOCX)

S5 Table Four African wild dog packs’ distances to different private nature reserves in the Limpopo and Mpumalanga provinces, South Africa, by availability and presence data, number of location points per feature subclass (n), feature subclass (by farm name), median distance (km), confidence interval (CI) and odds ratio. Odds ratios (ORs) were calculated as the difference between availability data and presence data (thus presence ORs = n/a) and indicate the probability of occurrence of a wild dog pack at any given agricultural feature subclass. OR = 1 indicates equal chance of occurrence, OR < 1 indicates low chance of occurrence and OR > 1 indicates high chance of occurrence.
(DOCX)
