# Supplementary material for: Landscape correlates of space use in the critically endangered African wild dog Lycaon pictus
Source: PLoS One. 2019 Mar 22;14(3):e0212621. doi: 10.1371/journal.pone.0212621 (PMC6430604; doi:10.1371/journal.pone.0212621)
Supplement: S1 Table — Odds ratios (ORs) were calculated as the difference between availability data and presence data (thus presence ORs = n/a) and indicate the probability of occurrence of a wild dog pack at any given agricultural feature subclass. OR = 1 indicates equal chance of occurrence, OR < 1 indicates low chance of occurrence and OR > 1 indicates high chance of occurrence. (DOCX) [file pone.0212621.s001.docx]

|  | | | | | | |
| --- | --- | --- | --- | --- | --- | --- |
| Pack | Status | n | Feature subclass | Median | CI | Odds Ratio |
| Waterberg | Available | 153 | Cattle | 0.33 | 0.74 | 1.52 |
|  |  |  |  |  |  |  |
|  |  | 116 | Goat | 0.00 | 1.20 | 0.66 |
|  |  | 130 | Poultry | 2.91 | 19.25 | 1.09 |
|  | Presence | 55 | Cattle | 0.46 | 0.73 | n/a |
|  |  | 257 | Goat | 0.00 | 0.06 | n/a |
|  |  | 87 | Poultry | 0.02 | 0.25 | n/a |
| Bluebank | Available | 551 | Cattle | 7.93 | 0.90 | 1.11 |
|  |  | 482 | Goat | 1.26 | 0.21 | 0.76 |
|  |  | 423 | Poultry | 4.67 | 4.06 | 0.90 |
|  | Presence | 607 | Cattle | 0.94 | 0.36 | n/a |
|  |  | 376 | Goat | 0.74 | 0.16 | n/a |
|  |  | 473 | Poultry | 0.06 | 0.02 | n/a |

S1 Table
