# Supplementary material for: Landscape correlates of space use in the critically endangered African wild dog Lycaon pictus
Source: PLoS One. 2019 Mar 22;14(3):e0212621. doi: 10.1371/journal.pone.0212621 (PMC6430604; doi:10.1371/journal.pone.0212621)
Supplement: S2 Table — Odds ratios (ORs) were calculated as the difference between availability data and presence data (thus presence ORs = n/a) and indicate the probability of occurrence of a wild dog pack at any given agricultural feature subclass. OR = 1 indicates equal chance of occurrence, OR < 1 indicates low chance of occurrence and OR > 1 indicates high chance of occurrence. (DOCX) [file pone.0212621.s002.docx]

S2 Table

|  | | | | | | |
| --- | --- | --- | --- | --- | --- | --- |
| Pack | Status | n | Feature subclass | Median | CI | Odds Ratio |
| Waterberg | Available | 371 | General farm | 10.27 | 0.84 | 1.78 |
|  |  | 13 | Goat farm | 3.04 | 1.08 | 0.56 |
|  |  | 15 | Hunting lodge | 10.81 | 1.68 | 1.01 |
|  | Presence | 395 | General farm | 7.73 | 0.48 | n/a |
|  |  | 3 | Hunting lodge | 10.30 | 8.16 | n/a |
| Skukuza | Available | 16 | Fruits & nuts | 19.94 | 1.09 | 1.20 |
|  |  | 31 | Game farm | 26.49 | 2.47 | 1.54 |
|  |  | 206 | General farm | 16.46 | 1.25 | 1.63 |
|  |  | 51 | Poultry farm | 27.29 | 1.31 | 1.06 |
|  | Presence | 3 | Game farm | 30.91 | 4.25 | n/a |
|  |  | 297 | General farm | 19.95 | 0.82 | n/a |
|  |  | 4 | Poultry farm | 27.17 | 9.03 | n/a |
| Orpen | Available | 229 | Fruit farms | 10.77 | 0.73 | 1.01 |
|  |  | 263 | Poultry farm | 11.45 | 0.64 | 0.99 |
|  | Presence | 230 | Fruit farms | 44.25 | 0.99 | n/a |
|  |  | 263 | Poultry farm | 44.61 | 0.85 | n/a |
| Bluebank | Available | 1431 | Fruit farms | 14.54 | 0.34 | 1.42 |
|  |  | 25 | Game farm | 20.76 | 0.53 | 0.70 |
|  | Presence | 1456 | Fruit farms | 5.00 | 0.24 | n/a |
