# Supplementary material for: Landscape correlates of space use in the critically endangered African wild dog Lycaon pictus
Source: PLoS One. 2019 Mar 22;14(3):e0212621. doi: 10.1371/journal.pone.0212621 (PMC6430604; doi:10.1371/journal.pone.0212621)
Supplement: S3 Table — Odds ratios (ORs) were calculated as the difference between availability data and presence data (thus presence ORs = n/a) and indicate the probability of occurrence of a wild dog pack at any given agricultural feature subclass. OR = 1 indicates equal chance of occurrence, OR < 1 indicates low chance of occurrence and OR > 1 indicates high chance of occurrence. (DOCX) [file pone.0212621.s003.docx]

S3 Table

|  | | | | | | |
| --- | --- | --- | --- | --- | --- | --- |
| Pack | Status | n | Feature subclass | Median | CI | Odds Ratio |
| Waterberg | Available | 14 | motorway | 4.99 | 1.90 | 1.09 |
|  |  | 13 | motorway link | 6.32 | 2.35 | 1.03 |
|  |  | 47 | primary | 2.89 | 1.28 | 0.91 |
|  |  | 42 | residential | 3.42 | 1.25 | 1.06 |
|  |  | 68 | secondary | 3.25 | 0.92 | 0.90 |
|  |  | 20 | tertiary | 3.97 | 2.96 | 0.93 |
|  |  | 56 | track | 2.92 | 1.42 | 0.90 |
|  |  | 34 | trunk | 1.95 | 1.33 | 0.83 |
|  |  | 5 | trunk link | 2.53 | 4.01 | 0.77 |
|  |  | 100 | unclassified | 2.79 | 0.84 | 0.91 |
|  | Presence | 22 | motorway | 5.34 | 1.76 | n/a |
|  |  | 13 | motorway link | 6.93 | 2.98 | n/a |
|  |  | 53 | primary | 4.19 | 1.07 | n/a |
|  |  | 34 | residential | 7.23 | 1.48 | n/a |
|  |  | 93 | secondary | 4.30 | 0.76 | n/a |
|  |  | 8 | service | 5.53 | 4.41 | n/a |
|  |  | 31 | tertiary | 2.90 | 1.05 | n/a |
|  |  | 16 | track | 6.19 | 1.09 | n/a |
|  |  | 38 | trunk | 3.33 | 1.18 | n/a |
|  |  | 5 | trunk link | 3.04 | 2.66 | n/a |
|  |  | 86 | unclassified | 4.21 | 0.87 | n/a |
| Skukuza | Available | 65 | secondary | 0.20 | 3.71 | 1.09 |
|  |  | 83 | tertiary | 9.86 | 3.32 | 0.92 |
|  |  | 44 | track | 37.14 | 7.29 | 1.08 |
|  |  | 112 | unclassified | 0.25 | 1.81 | 1.00 |
|  | Presence | 55 | secondary | 1.97 | 0.41 | n/a |
|  |  | 22 | service | 2.39 | 0.58 | n/a |
|  |  | 75 | tertiary | 1.23 | 0.39 | n/a |
|  |  | 13 | track | 0.54 | 0.79 | n/a |
|  |  | 139 | unclassified | 1.14 | 0.37 | n/a |
| Orpen | Available | 19 | motorway | 1.81 | 0.96 | 1.05 |
|  |  | 20 | motorway link | 1.86 | 1.29 | 1.04 |
|  |  | 56 | primary | 2.41 | 0.56 | 1.07 |
|  |  | 93 | residential | 1.71 | 0.46 | 1.05 |
|  |  | 93 | secondary | 1.66 | 0.49 | 0.92 |
|  |  | 28 | tertiary | 1.07 | 0.97 | 0.92 |
|  |  | 43 | track | 1.30 | 0.62 | 0.94 |
|  |  | 36 | trunk | 1.49 | 0.68 | 0.97 |
|  |  | 6 | trunk link | 2.01 | 3.34 | 1.05 |
|  |  | 98 | unclassified | 1.45 | 0.45 | 1.06 |
|  | Presence | 2 | residential | 0.92 | 0.87 | n/a |
|  |  | 91 | secondary | 1.46 | 0.33 | n/a |
|  |  | 130 | track | 1.48 | 0.32 | n/a |
|  |  | 270 | unclassified | 2.95 | 0.33 | n/a |
| Bluebank | Available | 20 | footway | 1.41 | 0.89 | 0.82 |
|  |  | 20 | motorway | 1.69 | 0.63 | 1.00 |
|  |  | 57 | motorway link | 1.37 | 0.47 | 1.13 |
|  |  | 12 | path | 1.36 | 0.89 | 1.03 |
|  |  | 83 | primary | 1.51 | 0.48 | 1.14 |
|  |  | 611 | residential | 1.64 | 0.16 | 1.20 |
|  |  | 134 | secondary | 1.56 | 0.26 | 1.12 |
|  |  | 65 | tertiary | 1.99 | 0.51 | 1.17 |
|  |  | 169 | track | 1.79 | 0.31 | 1.22 |
|  |  | 57 | trunk | 1.49 | 0.55 | 1.12 |
|  |  | 228 | unclassified | 1.59 | 0.26 | 1.20 |
|  | Presence | 16 | footway | 0.78 | 0.86 | n/a |
|  |  | 20 | motorway | 0.45 | 0.34 | n/a |
|  |  | 57 | motorway link | 1.33 | 0.38 | n/a |
|  |  | 12 | path | 0.76 | 0.71 | n/a |
|  |  | 83 | primary | 1.13 | 0.30 | n/a |
|  |  | 611 | residential | 1.34 | 0.11 | n/a |
|  |  | 138 | secondary | 1.16 | 0.23 | n/a |
|  |  | 65 | tertiary | 0.67 | 0.39 | n/a |
|  |  | 169 | track | 1.37 | 0.21 | n/a |
|  |  | 57 | trunk | 0.51 | 0.36 | n/a |
|  |  | 228 | unclassified | 1.37 | 0.18 | n/a |
