# Supplementary material for: Landscape correlates of space use in the critically endangered African wild dog Lycaon pictus
Source: PLoS One. 2019 Mar 22;14(3):e0212621. doi: 10.1371/journal.pone.0212621 (PMC6430604; doi:10.1371/journal.pone.0212621)
Supplement: S4 Table — Odds ratios (ORs) were calculated as the difference between availability data and presence data (thus presence ORs = n/a) and indicate the probability of occurrence of a wild dog pack at any given agricultural feature subclass. OR = 1 indicates equal chance of occurrence, OR < 1 indicates low chance of occurrence and OR > 1 indicates high chance of occurrence. (DOCX) [file pone.0212621.s004.docx]

S4 Table

|  | | | | | | |
| --- | --- | --- | --- | --- | --- | --- |
| Pack | Status | n | Feature subclass | Median | CI | Odds Ratio |
| Waterberg | Available | 8 | cemetery | 28.99 | 15.79 | 1.04 |
|  |  | 15 | commercial | 30.57 | 6.84 | 0.96 |
|  |  | 30 | industrial | 31.05 | 4.06 | 1.01 |
|  |  | 2 | landfill | 51.16 | 48.60 | 1.39 |
|  |  | 5 | military | 20.02 | 13.94 | 0.82 |
|  |  | 2 | quarry | 14.37 | 116.72 | 0.81 |
|  |  | 4 | railway | 16.39 | 18.24 | 0.81 |
|  |  | 241 | recreational | 32.67 | 1.59 | 1.03 |
|  |  | 24 | reservoir | 25.82 | 5.53 | 0.96 |
|  |  | 17 | residential | 26.05 | 7.50 | 1.05 |
|  |  | 51 | retail | 22.47 | 3.53 | 0.93 |
|  | Presence | 26 | cemetery | 32.82 | 4.11 | n/a |
|  |  | 15 | commercial | 34.78 | 4.79 | n/a |
|  |  | 30 | industrial | 31.65 | 3.21 | n/a |
|  |  | 2 | landfill | 35.76 | 37.74 | n/a |
|  |  | 5 | military | 36.27 | 15.14 | n/a |
|  |  | 2 | quarry | 44.63 | 68.88 | n/a |
|  |  | 4 | railway | 34.32 | 4.52 | n/a |
|  |  | 241 | recreational | 35.98 | 1.00 | n/a |
|  |  | 22 | reservoir | 36.77 | 3.46 | n/a |
|  |  | 11 | residential | 39.25 | 3.94 | n/a |
|  |  | 41 | retail | 34.51 | 3.41 | n/a |
| Skukuza | Available | 11 | cemetery | 2.08 | 4.51 | 0.85 |
|  |  | 12 | commercial | 9.44 | 4.46 | 1.17 |
|  |  | 20 | industrial | 10.66 | 2.72 | 1.14 |
|  |  | 2 | military | 11.98 | 47.80 | 1.33 |
|  |  | 2 | railway | 6.05 | 76.93 | 1.04 |
|  |  | 183 | recreational | 7.62 | 0.90 | 1.17 |
|  |  | 24 | reservoir | 9.16 | 2.31 | 1.22 |
|  |  | 5 | residential | 3.67 | 1.93 | 0.75 |
|  |  | 43 | retail | 6.93 | 2.22 | 1.18 |
|  | Presence | 15 | cemetery | 6.95 | 3.37 | n/a |
|  |  | 16 | commercial | 4.40 | 3.97 | n/a |
|  |  | 20 | industrial | 5.57 | 2.35 | n/a |
|  |  | 2 | military | 8.69 | 68.65 | n/a |
|  |  | 2 | quarry | 13.54 | 98.92 | n/a |
|  |  | 2 | railway | 9.74 | 7.99 | n/a |
|  |  | 215 | recreational | 8.73 | 0.70 | n/a |
|  |  | 22 | reservoir | 12.18 | 2.52 | n/a |
|  |  | 5 | residential | 1.11 | 10.46 | n/a |
|  |  | 3 | retail | 3.29 | 2.28 | n/a |
| Bluebank | Available | 1195 | military | 15.37 | 0.49 | 0.77 |
|  |  | 261 | reservoir | 20.87 | 0.66 | 1.29 |
|  | Presence | 1456 | military | 10.35 | 0.18 | n/a |
