# Supplementary material for: Landscape correlates of space use in the critically endangered African wild dog Lycaon pictus
Source: PLoS One. 2019 Mar 22;14(3):e0212621. doi: 10.1371/journal.pone.0212621 (PMC6430604; doi:10.1371/journal.pone.0212621)
Supplement: S5 Table — Odds ratios (ORs) were calculated as the difference between availability data and presence data (thus presence ORs = n/a) and indicate the probability of occurrence of a wild dog pack at any given agricultural feature subclass. OR = 1 indicates equal chance of occurrence, OR < 1 indicates low chance of occurrence and OR > 1 indicates high chance of occurrence. (DOCX) [file pone.0212621.s005.docx]

S5 Table

|  | | | | | | |
| --- | --- | --- | --- | --- | --- | --- |
| Pack | Status | n | Feature subclass | Median | CI | Odds Ratio |
| Waterberg | Available | 24 | Alem | 8.86 | 2.04 | 0.55 |
|  |  | 30 | Belanie | 5.96 | 1.45 | 0.60 |
|  |  | 8 | Geelhoutkop | 5.85 | 2.47 | 1.07 |
|  |  | 23 | Hen Nel | 1.55 | 1.38 | 0.56 |
|  |  | 92 | Innes Mellet | 9.72 | 1.44 | 1.24 |
|  |  | 20 | Kindjie | 5.33 | 1.23 | 1.08 |
|  |  | 36 | Les Brown | 6.25 | 1.17 | 0.92 |
|  |  | 5 | Ons Toekoms | 0.00 | 3.20 | 0.38 |
|  |  | 12 | Pearson | 1.18 | 1.28 | 0.68 |
|  |  | 60 | Pierre Fourie | 6.63 | 1.11 | 1.19 |
|  |  | 12 | Rinda | 4.54 | 1.78 | 0.71 |
|  |  | 54 | Rousseau | 9.85 | 1.39 | 1.30 |
|  |  | 10 | Van Heerden | 11.65 | 1.03 | 1.45 |
|  |  | 13 | Wyn Van Staden | 3.44 | 1.19 | 0.65 |
|  | Presence | 5 | Alem | 0.00 | 3.16 | n/a |
|  |  | 36 | Belanie | 0.00 | 0.49 | n/a |
|  |  | 80 | Hen Nel | 0.86 | 0.64 | n/a |
|  |  | 31 | Innes Mellet | 4.13 | 2.13 | n/a |
|  |  | 67 | Kindjie | 5.69 | 0.47 | n/a |
|  |  | 33 | Pearson | 3.10 | 0.83 | n/a |
|  |  | 134 | Pierre Fourie | 6.73 | 0.49 | n/a |
|  |  | 13 | Rousseau | 13.23 | 1.71 | n/a |
| Skukuza | Available | 304 | Sabie Sands | 26.28 | 1.37 | 1.18 |
|  | Presence | 304 | Sabie Sands | 18.71 | 0.97 | n/a |
| Orpen | Available | 13 | Cronje en Dogters | 0.00 | 0.28 | 0.73 |
|  |  | 3 | Giraffe | 0.39 | 1.21 | 2.19 |
|  |  | 30 | Klaserie | 0.00 | 0.20 | 0.74 |
|  |  | 112 | Manyeleti | 9.60 | 1.45 | 12.50 |
|  |  | 2 | Thornybush | 1.45 | 9.55 | 5.21 |
|  |  | 265 | Timbavati | 2.84 | 0.98 | 6.80 |
|  |  | 67 | Umbabat | 13.73 | 2.72 | 12.44 |
|  | Presence | 10 | Klaserie | 0.00 | 0.28 | n/a |
|  |  | 44 | Manyeleti | 16.31 | 1.60 | n/a |
|  |  | 438 | Timbavati | 6.27 | 0.50 | n/a |
| Bluebank | Available | 30 | Amsterdam | 0.00 | 0.30 | 3.93 |
|  |  | 459 | Bluebank | 8.54 | 0.58 | 11.79 |
|  |  | 89 | Blyolifant | 2.31 | 0.42 | 7.34 |
|  |  | 35 | Brussel | 0.67 | 0.40 | 3.83 |
|  |  | 88 | Kapama | 0.28 | 0.19 | 3.05 |
|  |  | 142 | Klaserie | 0.00 | 0.15 | 2.26 |
|  |  | 216 | Lissabon | 5.78 | 0.62 | 10.57 |
|  |  | 32 | Maroelanie | 1.55 | 0.45 | 4.59 |
|  |  | 129 | P. W. Willis | 1.30 | 0.48 | 5.530241 |
|  |  | 43 | Thornybush | 1.73 | 0.40 | 5.785112 |
|  |  | 101 | Vienna | 0.51 | 0.38 | 4.207169 |
|  |  | 65 | Welverdiend | 0.34 | 0.30 | 10.44736 |
|  |  | 27 | York | 0.27 | 0.32 | 2.328725 |
|  | Presence | 599 | Bluebank | 0.85 | 0.11 | n/a |
|  |  | 9 | Blyolifant | 2.59 | 0.44 | n/a |
|  |  | 59 | Kapama | 0.00 | 0.16 | n/a |
|  |  | 8 | Lissabon | 2.96 | 1.20 | n/a |
|  |  | 40 | P. W. Willis | 0.19 | 0.55 | n/a |
|  |  | 51 | Vienna | 0.00 | 0.26 | n/a |
|  |  | 690 | Welverdiend | 1.64 | 0.09 | n/a |
